# Achieving Ultrahigh Output Energy Density of Triboelectric Nanogenerators in High‐Pressure Gas Environment

**DOI:** 10.1002/advs.202001757

**Published:** 2020-11-17

**Authors:** Jingjing Fu, Guoqiang Xu, Changheng Li, Xin Xia, Dong Guan, Jian Li, Zhengyong Huang, Yunlong Zi

**Affiliations:** ^1^ Department of Mechanical and Automation Engineering The Chinese University of Hong Kong Shatin, N.T. Hong Kong SAR China; ^2^ Shun Hing Institute of Advanced Engineering The Chinese University of Hong Kong Shatin, N.T. Hong Kong SAR China; ^3^ State Key Laboratory of Power Transmission Equipment and System Security and New Technology School of Electrical Engineering Chongqing University Chongqing 401331 China

**Keywords:** breakdown voltage, electrical breakdown, high pressure gas, output energy density, surface charge density, triboelectric nanogenerators

## Abstract

Through years of development, the triboelectric nanogenerator (TENG) has been demonstrated as a burgeoning efficient energy harvester. Plenty of efforts have been devoted to further improving the electric output performance through material/surface optimization, ion implantation or the external electric circuit. However, all these methods cannot break through the fundamental limitation brought by the inevitable electrical breakdown effect, and thus the output energy density is restricted. Here, a method for enhancing the threshold output energy density of TENGs is proposed by suppressing the breakdown effects in the high‐pressure gas environment. With that, the output energy density of the contact‐separation mode TENG can be increased by over 25 times in 10 atm than that in the atmosphere, and that of the freestanding sliding TENG can also achieve over 5 times increase in 6 atm. This research demonstrates the excellent suppression effect of the electric breakdown brought by the high‐pressure gas environment, which may provide a practical and effective technological route to promote the output performance of TENGs.

## Graphical abstract

As an emerging high‐efficiency energy harvester, the output performance of the triboelectric nanogenerator (TENG) was limited by the inevitable electrical breakdown effect. A method for suppressing the breakdown effect in the high‐pressure gas environment is proposed here, which may provide a practical and effective technological route to promote the output performance of TENGs.

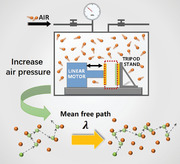

## 1 Introduction

In the 21st century, human society has experienced a new generation of information revolution. The interconnection of all things, informatization, and intelligence has become development trends, which rely on efficient energy system. To meet the rapidly increased energy demands, a new type of energy harvesting technology, nanogenerator, has been invented to provide sustainable power source by collecting energy from the ambient environment, which is based on the coupling of triboelectrification and electrostatic induction effects.^[^ ^1^ ^]^ This emerging technology was predicted to play a critical role in harvesting low‐frequency energy such as the body motion energy and ocean‐wave energy.^[^ ^2^ ^]^ Due to its advantages of lightweight, low cost, and high‐efficiency, plenty of research have demonstrated the great potential of TENGs on numerous applications.^[^ ^3^ ^]^

In order to promote the commercial development and widen application scenarios of TENG, intensive efforts have been focused on how to promote the output performance through various methods, such as structure optimization,^[^ ^4^ ^]^ material modification,^[^ ^5^ ^]^ surface treatment,^[^ ^6^ ^]^ the external electric circuit,^[^ ^3^, ^7^ ^]^ and so on. Although the surface charge density can be greatly enhanced up to be mC m^−2^ level by these methods, the breakdown threshold voltage still greatly limits the output energy density^[^ ^8^ ^]^ due to the electric breakdown, no matter which kinds of optimized methods were conducted. In order to quantitatively describe this output limit, researchers introduced a new standardized evaluation method^[^ ^9^ ^]^ to calculate the effective maximized output energy *E*_em_ per cycle as limited by the breakdown effect,^[^ ^10^ ^]^ which provides a key parameter to evaluate TENGs′ performance as an energy harvester. *E*_em_ is mostly limited by the gas breakdown in the gap between electrodes and/or tribo‐surfaces which is evitable in TENGs, which thus can be adjusted by the gas pressure. Previously Wang et al. demonstrated an effective method through ultrahigh vacuum to greatly promote the output power density by ≈6–7 times by suppressing the gas breakdown effect.^[^ ^11^ ^]^ However, this boosting effect can only work at ultrahigh vacuum. When the vacuum is below ultrahigh level, for example in the range of 100 to 800 Torr, TENG's output performance does not increase but obviously decreases due to the boosted breakdown effect.^[^ ^12^ ^]^ This issue raises many higher demands on the ultra‐high‐vacuum packaging of the devices. For example, the environment of 10^−8^ Torr should be vacuumed by a turbomolecular pump^[^ ^13^ ^]^ and be measured by an ionization vacuum gauge^[^ ^14^ ^]^ or a magnetron vacuum gauge.^[^ ^15^ ^]^ In the meanwhile, how to maintain the ultrahigh vacuum environment makes a great challenge on the packaging technology.

As a more practical way to achieve performance enhancement beyond the breakdown limit, this report proposes the high‐pressure gas environment to suppress the breakdown effect. The increased gas pressure causes the shorter mean free path and thereby inhibits the gas breakdown. This report demonstrated the enhancement of the threshold charge density *σ*_T_ and the threshold open‐circuit voltage *V*_TOC_ with the increased gas pressure. Moreover, we demonstrated that the maximum output energy density can be increased by over 25 times for contact‐separation mode (CS) TENG and 5 times for sliding‐freestanding mode (SFT) TENG. As compared, the high‐pressure gas proposed in our method is more common in our daily life, such as that in tires, cylinders, and engines, with much mature technologies. Besides, for ocean energy harvesting, the high pressure in the packaged TENG can well balance the high water pressure in the deep ocean environment, making it a perfect design toward the “blue energy” dream.^[^ ^16^ ^]^This research provides a practical and effective technological route to promote the output performance of TENG beyond the breakdown limit, which will lay the foundations in further applications and the industrialization of TENG technology in the future.

## 2 Results

### 2.1 The Working Principle and Experimental Setups of the Gas Breakdown Suppression by High‐Pressure Gas Environment

**Figure 1:**
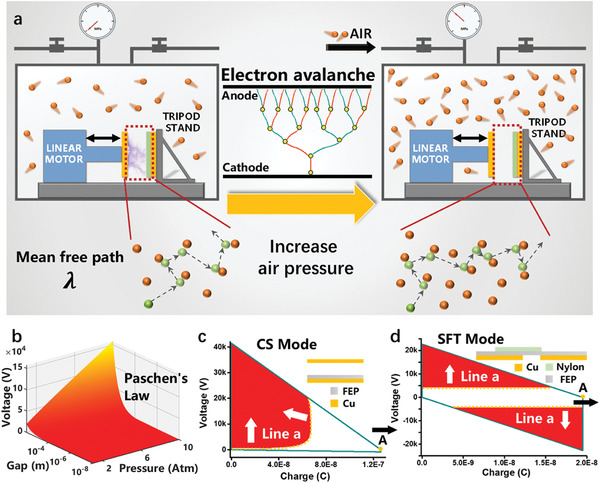
The working principle of suppressing gas breakdown in TENGs by the high‐pressure gas environment to promote the output energy density of TENG. a) The schematic of experiment setups and the working principle of suppressing gas breakdown by the high‐pressure gas environment in TENG. b) The 3D diagram shows the relationship among the breakdown voltage, air gap distance, and pressure. (c) and (d) show the *V–Q* plots of CS mode TENG and SFT mode TENG showing the gas breakdown suppression effect, respectively.

The gas breakdown effect is inevitable in TENGs due to air gaps between two tribo‐surfaces or two electrodes, which follows Townsend's electron avalanche model,^[^ ^17^ ^]^ as shown in the middle inset of **Figure** **1**a. When the voltage surpasses the threshold breakdown voltage, *V*_b_, or the electric field exceeds the threshold breakdown electric field, electrons emitted from the cathode will be accelerated to a high kinetic energy, which will constantly collide with the gas molecules and cause collision ionization. And then the secondary electrons will be formed during the collision to initiate the chain reaction, as called electron avalanche.^[^ ^18^ ^]^ Such collision ionization can only be formed when the kinetic energy of electrons, *eEx*, exceeds the ionization energy of gas molecules, *W_i_*:

(1) $$\begin{array}{c} eEx\geq W_{i} \end{array}$$

Here, *x* is the distance of an electron travels in an electric field before hitting any gas molecules; *e* is the charge of an electron, 1.6 × 10^−19^ C; *E* is the electric field strength. Usually we use the mean free path, *λ*, instead of *x* to determine whether the breakdown will happen or not. Thus, with the same electric field strength, reducing the mean free path of electrons can suppress the breakdown effect.

From the perspective of reducing the mean free path of electrons, increasing gas pressure of the working environment for TENG is an effective method, as shown in Figure 1a, and thus the air breakdown effect is suppressed. For the parallel‐plate capacitor model, the enhancement of *V*_b_ can be directly calculated by Paschen's law,^[^ ^17c^ ^]^ as shown in Equation (2). (The parameters used in this equation shown in Table S1, Supporting Information)

(2) $$\begin{array}{c} V_{b}=\frac{Bpd}{\ln\left(Apd\right)-\ln\left(\ln\left(1+\frac{1}{\gamma_{\mathrm{se}}}\right)\right)} \end{array}$$

Figure 1b shows the relationship between *V*_b_ and the distance *d* of the air gap and gas pressure *p*, simultaneously. Obviously, with the increase of pressure the breakdown voltage improves. Especially when *d* is larger, the enhancement of *V*_b_ is more obvious. Besides the breakdown voltage, the threshold charge density of TENG is also influenced by gas pressure, as determined by the breakdown effect in the short‐circuit condition.^[^ ^11^ ^]^ These two threshold values together confine *E*_em_ in the *V*–*Q* plot, which determines the maximized output energy density,^[^ ^9^, ^10^ ^]^ as demonstrated in Figure 1c,d. Herein, we first designed the experimental setups shown in Figure 1a to provide a high‐pressure gas environment to promote the output energy density. With the linear motor setups inside the high‐pressure chamber and measurement circuits developed previously,^[^ ^10^, ^19^ ^]^ we developed the capabilities to measure the threshold voltage and charge outputs against the breakdown effect, which can be used to calculate the max output energy density.

### 2.2 The Threshold Open‐Circuit Voltage of TENGs

**Figure 2:**
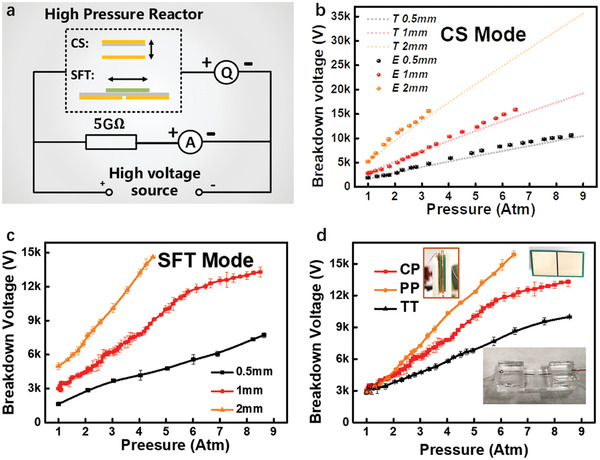
The threshold open‐circuit voltage *V*_TOC_ measurement of TENGs. a) The circuit diagram for the experiment. b) The theoretical (T) and experimental (E) *V*_TOC_ of the CS‐TENG under various pressure and gap distance. c) The experimental *V*_TOC_ of the SFT‐TENG under various pressure and gap distance. d) The experimental *V*_TOC_ of the various electrode shapes under various pressure and the identical gap distance. Each point in Figure (b), (c), and (d) was tested for 3–5 times.

As developed by the previous studies, the voltage *V* and charge transfer *Q* can be measured simultaneously to reflect the impact of the air pressure on the breakdown points, through the circuit as shown in **Figure** **2**a.^[^ ^10^, ^19^ ^]^ As shown in Figure S1 and Note S1, Supporting Information, when the breakdown happens, there will be an obvious turning point in the measured *V*–*Q* curve, while the slope of the linear part before the turning point is reciprocal of the capacitance. We firstly measured the threshold open‐circuit voltage *V*_TOC_ at the target gas pressure achieved by adding the dry air into the reactor containing a CS‐TENG. This *V*_TOC_ was measured by using the linear motor to trigger the separation process under the open‐circuit condition. To verify the experimental results, we also conducted the theoretical simulations through finite‐element method (FEM), with detailed method described in Note S2 and Figure S2, Supporting Information. Apparently the experimental measured *V*_TOC_ fit with the theoretical results quite well, as shown in Figure 2b. Under different gap distances between two electrodes, the impact of the gas pressure was studied as illustrated in Figure 2b,c. As the gap increases, the *V*_TOC_ will increase as we considered that the threshold electric field keeps almost the same at the same pressure. According to these results, with the gas pressure of ≈5–6 atm, the increase in *V*_TOC_ can achieve 5–6 times. Supplementary Video 1 and Movie 2 demonstrate that higher voltage is required to induce the breakdown at the higher pressure. One thing should be noticed is that the gap and the breakdown voltage do not strictly satisfy the linear relationship, especially when the gas pressure is high. It is because when the pressure is high, the non‐uniform electric field caused by edge effects, surface roughness, and etc. will much lower the threshold voltages. Besides with the gap between two electrodes, the shape of electrodes can also affect the breakdown voltage, as shown in Figure 2d. The tip‐to‐tip (TT) electrodes were designed for comparing the *V*_TOC_ against that of CS‐TENG (parallel‐plane, or PP electrodes) and SFT‐TENG (co‐planar, or CP electrodes). According to the result in Figure 2d, we could find the sequence of *V*_TOC_, as follows:

(3) $$\begin{array}{c} \mathrm{PP}>\mathrm{CP}>\mathrm{TT} \end{array}$$

This sequence is in accordance with the dimension of the uniformity of their electric field, since PP, CP, and TT have 2D, 1D, and 0D uniformity, respectively. This observation may provide us a general guideline to design our device to suppress or enhance the effect of the air breakdown through the shape of electrodes. Herein, we can confirm that the *V*_TOC_ can be obviously improved by increasing the gas pressure, which means that the red areas as shown in *V*–*Q* plots in Figure 1c,d can be shrunk down. Here we extracted the capacitance from the non‐breakdown part in experimentally measured *V–Q* curves under the different gas pressures, as shown in Figure S3, Supporting Information. The detailed calculation progress is described in Note S1, Supporting Information. We can see that the capacitance of devices is almost invariant with the gas pressure, since the pressure has little influence on the dielectric constant of the gas.

### 2.3 The Threshold Charge Density of TENGs

Generally, the electric field of TENGs at the short‐circuit condition is obviously weaker than that at the open‐circuit condition, which could help the friction layers to accumulate more charge.^[^ ^20^ ^]^ While the threshold short‐circuit charge density *σ*_T_ still exists due to breakdown, as reported previously.^[^ ^11^, ^21^ ^]^ Taking the CS mode TENG as an example, the voltage of the gap between two tribo‐surfaces in the short‐circuit condition, *V*_1*SC*_, is given by:

(4) $$\begin{array}{c} V_{1sc}=\frac{t\sigma d}{\varepsilon_{0}\left(t+d\varepsilon_{r}\right)} \end{array}$$

When it surpasses the breakdown voltage calculated by Equation (1), the breakdown happens. Hence the threshold charge density *σ*_T_ can be derived by the following equation:

(5) $$\begin{array}{c} \sigma_{T}=\min\left[\frac{Bp\varepsilon_{0}\left(t+d\varepsilon_{r}\right)}{t\left(\ln\left(Apd\right)-\ln\left(\ln\left(1+\frac{1}{\gamma_{\mathrm{se}}}\right)\right)\right)}\right] \end{array}$$

**Figure 3:**
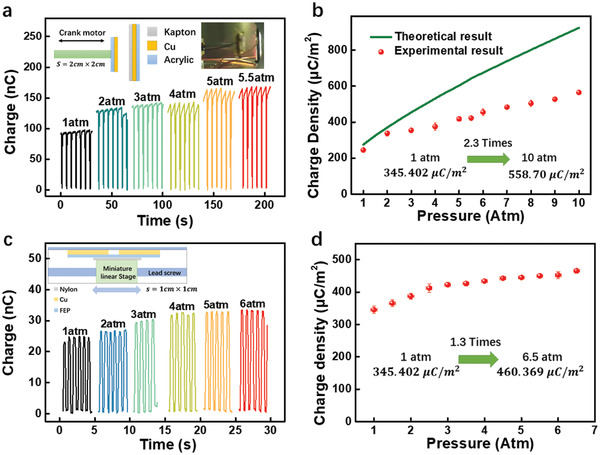
The threshold charge density of TENGs. a) The transferred charge of CS mode TENG at the short‐circuit condition. The insets are the schematic and the photo of the tested device. b) The theoretical and experimental *σ*_T_. c) The transferred charge of SFT mode TENG at short‐circuit condition. The insets are the schematic and the photo of the tested device. d) The theoretical and experimental *σ*_T_. Each point in Figure (b) and (d) was tested for 3 times.

The detailed derivation progress is shown in Note S3, Supporting Information. As shown in the green line in **Figure** **3**b, theoretically, the charge density can be boosted with the increase of the gas pressure, and there is a 2.5 multiple of growth at 6 atm.

To push the limit of *σ*_T_ under high‐pressure gas environment, self‐enhancing circuit^[^ ^7^ ^]^ was used to accumulate charge generation (see Figure S4 and Note S4, Supporting Information) and the material selection was optimized to resist the high pressure (see Figure S5, Supporting Information). From the experiment as illustrated in Figure 3a and Supplementary Movie 3, an increasing trend can also be found. Through the comparison of experimental and theoretical results in Figure 3b,c, it is shown that the experimental *σ*_T_ under relatively low pressure (≤2 atm) fits the calculated results well, while under high pressure the experimental *σ*_T_ is always much lower than the theoretical results. The difference between experimental and theoretical results may be due to the enhanced local electric field brought by the edge effects and the non‐ideal tribo‐surfaces with some surface roughness, which makes the breakdown prone to happen with high charge density.^[^ ^22^ ^]^

For SFT TENG, the breakdown condition is more complex and the theoretical model is still not mature. There could be various types of breakdown in this TENG, such as the breakdown between two electrodes, the dielectric breakdown in the middle friction layer and the breakdown between two tribo‐surfaces during sliding, as shown in Figure S6, Supporting Information, and hence the impact of the gas pressure is limited. At the open‐circuit condition, the major breakdown limit may be contributed by that between electrodes, due to the huge potential difference and the electric field strength. However, at the short‐circuit state, the potential of two electrodes keeps the same which makes the corresponding electric field strength much smaller, making the other two types of breakdown determine *σ*_T_. Considering that the dielectric breakdown does not rely on the gas pressure, the experimental *σ*_T_ undergoes a limited increase as shown in Figure 3c,d, and is obviously lower than the dielectric charge density limit, 6.1 mC m^−2^, which indicates there is a large space for optimizing the triboelectric charge density, which may be conducted through multiple existing methods.^[^ ^11^, ^23^ ^]^

### 2.4 The Output Energy Density of TENGs

**Figure 4:**
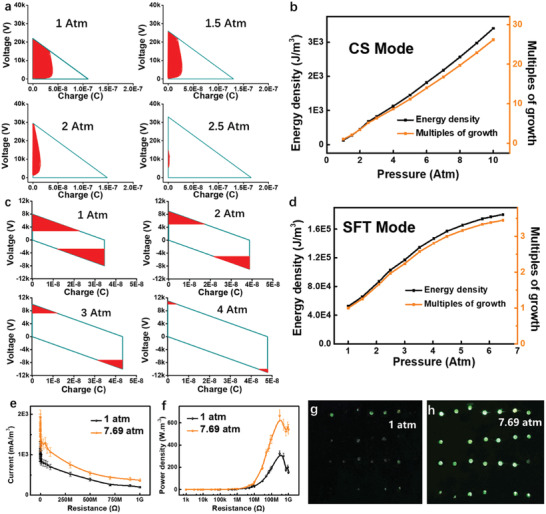
The output energy density TENGs under different pressure. a) The *V–Q* curves of the CS TENG at different gas pressure. b) The histogram of the energy density of CS TENG versus the gas pressure. The insert is the relationship between the multiples of growth of energy density and the pressure. c) The *V–Q* curves of the SFT TENG at different pressure. d) The histogram of the energy density of SFT TENG versus the gas pressure. The insert is the relationship between the multiples of growth of energy density and the pressure. (e) and (f) show the current and power of CS TENG (2 cm × 2 cm × 1 cm) with various loads at 1 atm and 7.69 atm. (g) and (h) demonstrate the matrix of 7 × 4 LEDs in series charged by CS TENG, 2 cm × 2 cm × 1 cm, at 1 atm and 7.69 atm, respectively. Each point in Figure (e) and (f) was tested for 3 times.

According to the previous report, *E*_em_ is defined as to the area of the non‐breakdown area in the *V–Q* curve of CMEO,^[^ ^9^ ^]^ which can be confined by *V*_TOC_ and *σ*_T_. Therefore, by using experimentally measured *V*_TOC_ and *σ*_T_ combining previous models,^[^ ^10^ ^]^ the breakdown lines can be determined in *V–Q* curves of CS and SFT TENGs at the various gas pressure as plotted in **Figure** **4**a,c, respectively. (See Note S5 and Figure S7a,b, Supporting Information, for detailed calculation methods) It's obvious that as the gas pressure increases, the non‐breakdown area in white enlarges. In order to illustrate and compare the output performance in a better way, the output energy density *U* is investigated,^[^ ^10b^ ^]^ given by:

(6) $$\begin{array}{c} U=\frac{E_{\mathrm{em}}}{V_{L}} \end{array}$$

Here, *V_L_* is the volume of the tested device. (The detailed calculation process is described in Note S5 and Figure S7c,d, Supporting Information.) Figure 4b,d shows the increase of the output energy density *U* and gas pressure *p*. From these results, there are amazing 25 multiplies of output energy density growth at 10 atm for CS TENG and 5 times increase at 6.5 atm for SFT TENG.

Furthermore, taking CS TENG of 2 cm × 2 cm as an example, the elevated output power in the high‐pressure gas environment is demonstrated through driving electronics. Here, the triboelectric layers of this CS TENG are the Cu and Kapton (50 µm), and its soft substrate is silicone, with the maximum separation distance *d*_m_ of 1cm. When this CS TENG is operated in the high gas‐pressure (7.69 atm), the breakdown effect is obviously suppressed and the peak short‐circuit current raises from 4.6 to 8 µA (Figure 4e). And the maximum power density is also enhanced from 324 to 664 W m^−3^ (Figure 4f) with the match load of 300 MΩ at a frequency of 1 Hz. Moreover, a bulb matrix of 7 × 4 LEDs in series as shown in Figure S8, Supporting Information, can be lit by TENG working in the high‐pressure gas environment (Figure 4h), which was demonstrated with much higher brightness compared with that operated in the atmosphere (Figure 4g), as shown in Supplementary Movie 5. This experiment clearly demonstrated the output performance of TENG could be obviously enhanced in the high‐pressure gas environment.

## 3 Discussion

In summary, we firstly demonstrated the boosted output energy density of TENGs in the high‐pressure gas environment to suppress the breakdown effect, through both theoretical and experimental studies. We measured the increase of the threshold open‐circuit voltage with the gas pressure for both CS and SFT mode TENGs. The great enhancement of output energy density was achieved with over 25 times for CS TENG at 10 atm and 5 times for SFT TENG at 6 atm, with boosted power output performance demonstrated. This study not only conducted a crucial fundamental investigation on the impact of the pressure on suppressing the breakdown effect, but also provided a practical and effective solution to greatly enhance the output performance of TENGs, with broad potential applications such as self‐powered sensing in high‐pressure environment and the ocean energy harvesting.

## 4 Experimental Section

##### Fabrication Device and Measurement Experiment

For the experiment to test the threshold open‐circuit voltage, the electrodes were fabricated on the printed electric board, Cu with 2 cm × 2 cm × 0.01 cm. The dielectric layer for CS was 50 µm Kapton. The middle layer for SFT TENG was 50 µm Kapton, and the top moving layer is 50 µm Nylon. The precise position was controlled by the combination of the fixed bracket and XYZ‐stage, with the minimum movement of 10 µm. The electric circuit diagram for the experiment is shown in Figure 2a. The transferred charge was measured by the Keithley 6514 electrometer. For the voltage, it was calculated by multiplying the resistance (5 GΩ) and the current transferred it measured by MODEL SR 570 (*V* = *I* × *R*). For the experiment to test the threshold charge density, use Cu (2000 Å) as the electrodes which is directly evaporated on the surface of Kapton film (50 µm) by the E‐beam evaporator. For CS TENG, in order to avoid the decrease of the contact area caused by misalignment, the two contact faces were designed with a different size. One side is the copper tape as the friction layer and electrode (2 cm × 2 cm), and the other side is using the Kapton film as the frication layer and using the Cu evaporated on the film as the electrode (3 cm × 3 cm).

##### Motor and Sealed Method

For achieving the linear movement in the internal chamber of a high‐pressure reactor without air leakage, a remotely controlled mini linear motor (145 mm × 23 mm × 23 mm) powered by a 12 V battery was used, instead of the large traditional linear motor with cables required. Therefore, this small linear motor can be set in the chamber with no cable/wire required, without the leakage issue. And besides, the wires used to collect signals for TENG measurement are very fine enameled copper wire. With the assistance of PDMS seal, there is no leakage issue induced as well.

## 5 Statistical Analysis

### 5.1 Pre‐Processing Progress

Original data measured by Keithley 6514 and MODEL SR 570 cannot be directly used in the following analysis. Usually, we first transferred the absolute time to relative time as data's *x* axils, to make it clearer. Then transfer the original data to the aimed one. For the experiment of measuring *V*_TOC_ (Figure 2b,c), the current should be multiplied with resistance to be transferred as voltage, = *I* × *R*. For the experiment of measuring *σ*_T_ (Figure 3c,d), the measured transferred charge should be divide by its area, *S*, to show as charge density, *σ*_T_ = *Q*_sc_ /*S*. For the experiment to get the power of CS TENG at different gas pressure (Figure 4f), power is calculated as *P* = *I* ^2^ × *R*. This process is completed by Microsoft Excel.

### 5.2 Errors in Experiments

First are errors from the measurement system. The digital display barometer used in this paper has a range (*R*) of 0–16 atm and an accuracy (*θ*) of 0.5%, (± 0.4 level). The measurement error (*E*_m_) can be calculated: *E*_m_ = *R* × *θ* = 0.08 atm. Second is the error from multiple measurements. This error may occur due to subtle differences in operation, which is tolerated and should be shown in the final result. But if the device is significantly damaged because of working for a long period, this type of data should be discarded. Like in the experiment to get *V*_TOC_, if the device has discharged for a long time, the surface of electrodes will be damaged and the data will be significantly away from the average value. It is worth noting that in the experiment to measure *V*_TOC_, even for the device measured in the same batch, *V*_TOC_ is also not exactly the same, as shown in Figure S1, Supporting Information. This is because the discharge location at each time is random and unpredictable, which may be related to the smoothness of the device edge, the flatness of the surface, and the subtle difference in the thickness of the electrode composition at each location. Such errors are also reasonable and need to be considered in the final result.

### 5.3 Final Data Presentation

Mean + standard deviation (Y error) + measurement error (X error). Sample size: For the experiment of *V*_TOC_ there are five groups of data tested at different times. For the experiment of *σ*_T_ and the current and power of CS TENG under different gas pressure, there are three groups of data measured at different times. Final results are processed and displayed by Origin (data analysis software).

## Conflict of Interest

The authors declare no conflict of interest.

## Supporting information

Suppoting Information

Supplemental Movie 1

Supplemental Movie 2

Supplemental Movie 3

Supplemental Movie 4

Supplemental Movie 5
