# Supplementary material for: Achieving Ultrahigh Output Energy Density of Triboelectric Nanogenerators in High‐Pressure Gas Environment
Source: Adv Sci (Weinh). 2020 Nov 17;7(24):2001757. doi: 10.1002/advs.202001757 (PMC7740098; doi:10.1002/advs.202001757)
Supplement: Supplementary file 1 — Suppoting Information [file ADVS-7-2001757-s001.pdf]

© 2020 Wiley-VCH GmbH

Supporting Information

for *Adv. Sci.*, DOI: 10.1002/advs.202001757

Achieving Ultrahigh Output Energy Density of Triboelectric Nanogenerator in High-Pressure Gas Environment

Jingjing Fu, Guoqiang Xu, Changheng Li, Xin Xia, Dong Guan, Jian Li, Zhengyong Huang, Yunlong Zi**

Supporting Information

Achieving Ultrahigh Output Energy Density of Triboelectric Nanogenerator in High-Pressure Gas Environment

Jingjing Fu, Guoqiang Xu, Changheng Li, Xin Xia, Dong Guan, Jian Li, Zhengyong Huang*, Yunlong Zi*

J. Fu, G. Xu, X. Xin, D. Guan, Prof. Y. Zi

Department of Mechanical and Automation Engineering

The Chinese University of Hong Kong

Shatin, N.T., Hong Kong SAR, China.

Email: ylzi@cuhk.edu.hk

J. Fu, Prof. Y. Zi

Shun Hing Institute of Advanced Engineering

The Chinese University of Hong Kong

Shatin, N.T., Hong Kong SAR, China.

Email: ylzi@cuhk.edu.hk

C. Li, Prof. J. Li, Dr. Z. Huang

State Key Laboratory of Power Transmission Equipment and System Security and New Technology

School of Electrical Engineering

Chongqing University

Chongqing 401331, China.

Email: huangzhengyong@cqu.edu.cn

ADVANCED SCIENCE
Open Access

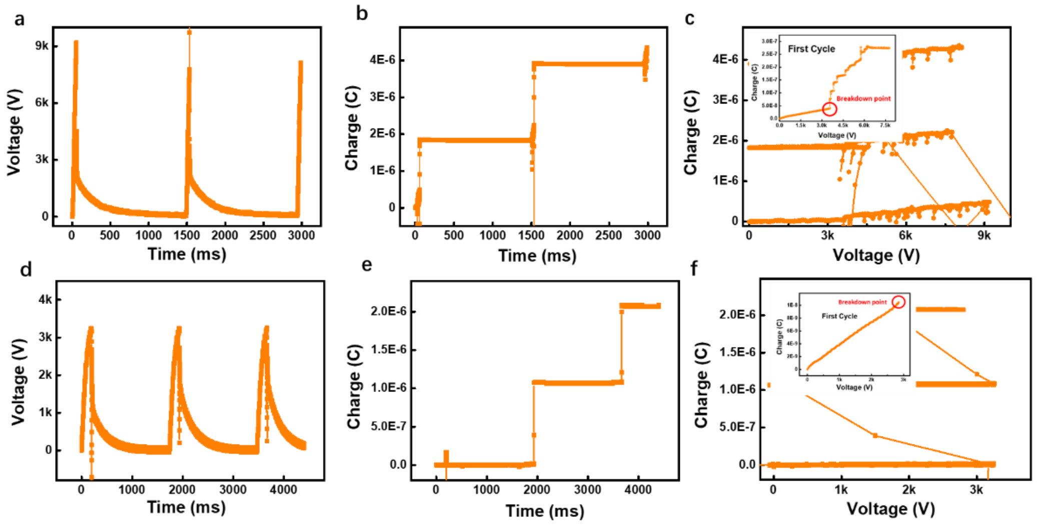

Figure S1 The experiment to measure the breakdown voltage of CS and SFT TENG. a) and d) are the voltage of the CS and SFT TENG. b) and e) are the transferred charge of the CS and SFT TENG. c) and f) show the V-Q-curve of TENGs, and the inserts show the first cycle of TENGs before the breakdown happens.

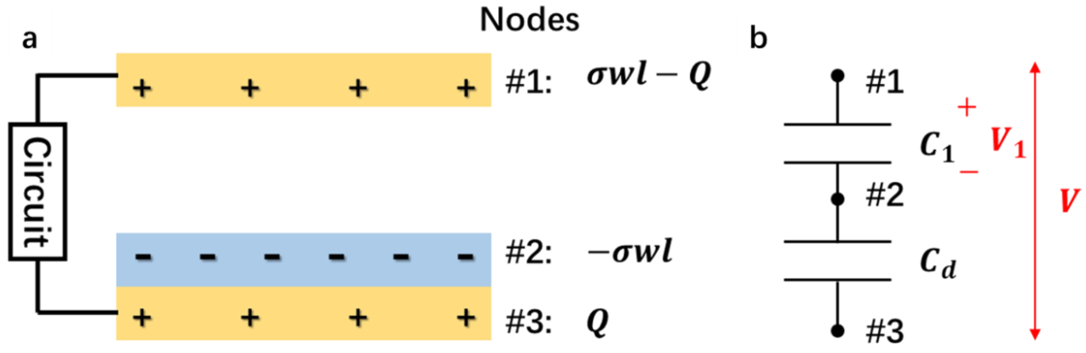

Figure S2 The working principle of the CS TENG. a) The schematic with nodes and the surface charge for CS TENG. b) The equivalent circuit diagram with the capacitance and the voltage for CS TENG.

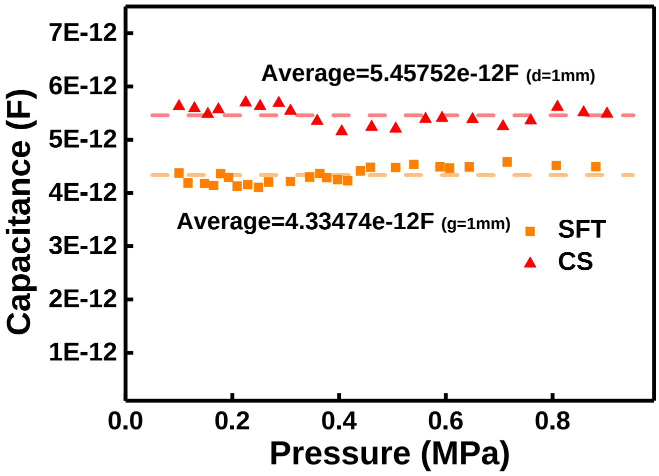

Figure S3 The capacitance of TENGs vs. the gas pressure. The points represent the experimental data. The dotted lines show the average capacitance for each experimental group.

ADVANCED SCIENCE Open Access

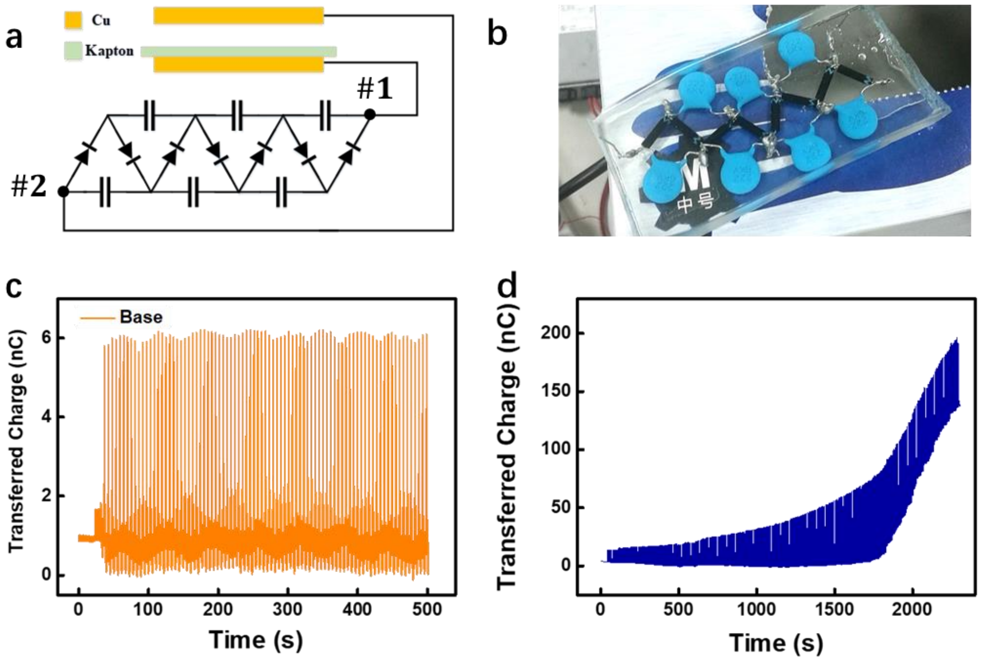

Figure S4 The method to achieve the higher transferred charge. a) shows the electric circuit diagram of the self-enhancing circuit used in this experiment. b) is the picture of the electric circuit encapsulated in insulated silicone. c) The transferred charge of the tested device without the self-enhancing circuit. d) The transferred charge of the tested device with the self-enhancing circuit.

ADVANCED SCIENCE
Open Access

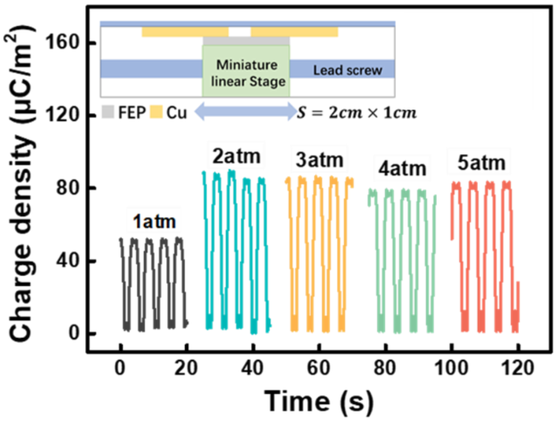

Figure S5 The charge density of the tested SFT TENG using as the foam as the soft base. The insert shows the working principle diagram of the device.

ADVANCED SCIENCE Open Access

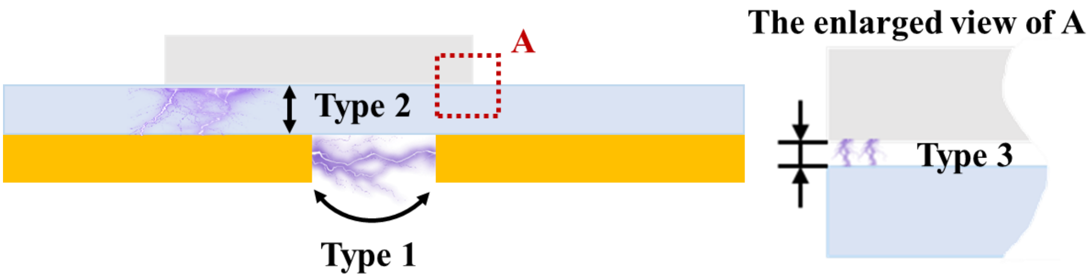

Figure S6 The breakdown of SFT TENG. Three breakdown type for SFT TENG. The right inset is the enlarged view for the Partial area A.

ADVANCED SCIENCE Open Access

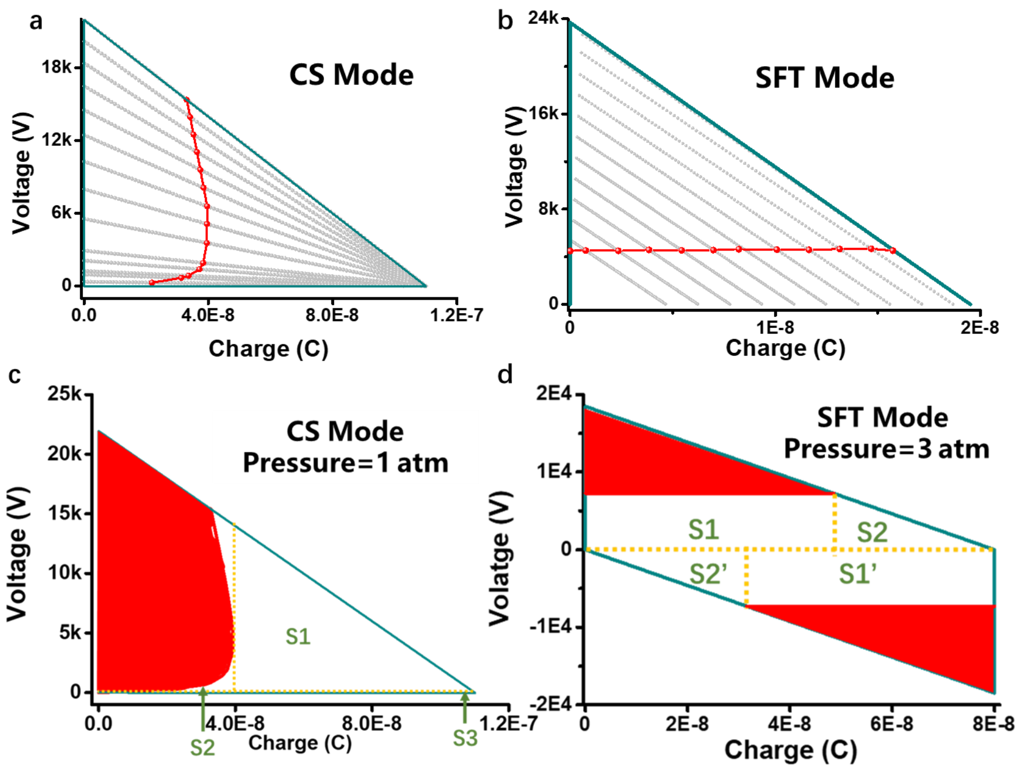

Figure S7 How to calculate the output energy of TENGs limited by the breakdown effect. a) and b) show the *V*-*Q* curves for CS and SFT mode TENG, respectively. In c) and d), the red part is the breakdown area. The yellow lines divided the white area into several small parts in order to calculate easily.

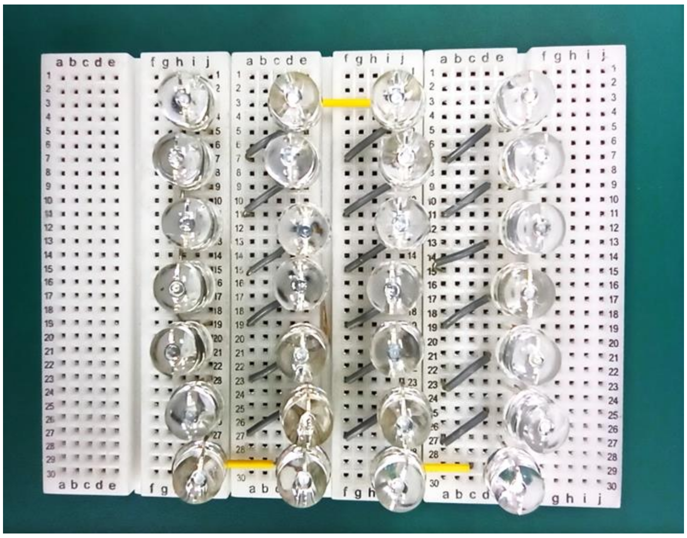

Figure S8 The picture of the matrix of 7×4 LEDs (0.25W×28) in series.

Table S1. Parameters used in CS TENG (FEP-Cu)

$\boldsymbol{A}[1/(atm.m)]$	$\boldsymbol{B}[V/(atm.m)]$	$\boldsymbol{\gamma_{se}}$	$\boldsymbol{\varepsilon_0}$	$\boldsymbol{\varepsilon_r}$	$\boldsymbol{d_r}(m)$	$\boldsymbol{w}(m)$	$\boldsymbol{l}(m)$
6475697	19046167	0.025	8.85419×10^{-12}	2.1	5×10^{-5}	0.02	0.02

Notes:

A and B are the constants determined by the composition of the gas.

γ_{se} is the secondary-electron-emission coefficient.

ε_0 is the vacuum dielectric permittivity.

ε_r is the dielectric permittivity of FEP.

d_r is the thickness of the FEP film.

w is the width of the device.

l is the length of the device.

Table S2. Parameters used in SFT TENG (Kapton-Nylon, Cu)

$E_b(V/m)$	ε_r	$d_r(m)$	$w(m)$	$l(m)$
2×10^8	3.4	5×10^{-5}	0.01	0.01

Notes:

E_b is the dielectric strength of Kapton.

ε_r is the dielectric permittivity of Kapton.

d_r is the thickness of the Kapton film.

w is the width of the device.

l is the length of the device.

Note S1 The experiment to get the breakdown voltage for CS and SFT TENG.

The circuit diagram of this experiment is shown in Figure 2a. We use the commercial high voltage power to charge the tested TENG, because of its stability and reliability. At the same time, due to its high-voltage and low-current output characteristics, the experimental process is safer than expected. By using the two-channel testing program in LabVIEW software, we can measure the transferred charge and the voltage of the TENG by KEITHLEY 6514 electrometer, simultaneously. It should be noticed that if we measure the voltage directly, it is so easy to overload. In order to solve this, we calculate the voltage by measuring the current through the big resistance instead of measurement the voltage directly, as shown in Figure 2a. Figure S1 shows the experimental data we got. When there was no breakdown, the charge will continue to increase with the increase of voltage, due to TENG's capacitance characteristic, as shown in the inserts of Figure S1c and S1f. The slope of this liner part is the capacitance of TENG, as shown in Figure S3. But when a breakdown happens, the voltage and the transferred will undergo great change, and the *V-Q* curve for the device will not be linear as before. There will be many random points in the *V-Q* curve, during the breakdown process, as shown in Figure S1c and S1f. Because of the limitation of the accuracy and range of the measuring equipment, the value of these random points may be not accurate. Therefore, the value of these points doesn't have practical significance. But the last point before the breakdown happens is pretty critical for us. This is the breakdown voltage and the breakdown charge of the tested device. Some previous researches have already confirmed this measurement method can be used in many types of device [1].

Note S2 Calculation of the breakdown voltage of CS TENG.

As shown in Figure S2, the CS TENG can be equalized as two capacitors in series connection. The first is the capacitor of the dielectric layer, and its capacitance can be calculated as [2]:

$$C_d = \varepsilon_0 \varepsilon_r \frac{lw}{d_r} \tag{S1}$$

The second one is the capacitor between two triboelectric layers. Its capacitance, charge and voltage can be calculated as following [2b, 3]:

$$C_1 = \varepsilon_0 \varepsilon_r \left\{ \frac{lw}{d} + \frac{l}{\pi}\left[1 + \ln(1 + 2\pi\frac{w}{d} + \ln(1 + 2\pi\frac{w}{d})\right] + \frac{w}{\pi}\left[1 + \ln(1 + 2\pi\frac{l}{d} + \ln(1 + 2\pi\frac{l}{d}))\right] \right\} \tag{S2}$$

$$Q_1 = \sigma \ w \ l \ (\text{[illegible]} \tag{S3}$$

$$V_1 = \frac{Q_1}{C_1} \tag{S4}$$

And then the total capacitance of CS TENG is:

$$C = \frac{C_1 \times C_d}{C_1 + C_d} \tag{S5}$$

The voltage of CS TENG is equal to:

$$V = \frac{Q}{C} \tag{S6}$$

After describing the calculation method of the simplified equivalent physical models, we can further calculate its breakdown voltage.

For CS TENG, the breakdown mainly happen in the air gap between two triboelectric layers, which means that it only happens when the voltage of capacitor 1 is over than the limit breakdown voltage calculated by the Paschen's, as shown in Equation (1)

$$V_1 \geq V_b \tag{S7}$$

Through this equation, we could get the limited breakdown voltage of C_1, and then according to the relationship between V_1 and V, as shown above, we can finally get the breakdown voltage of CS TENG.

Note S3 Calculation of the threshold charge density of CS TENGs

As mentioned above, for CS TENG, the breakdown mainly happens in the air gap between two triboelectric layers, and the voltage of this part can be calculated as:

$$V_{1SC} = \frac{t\sigma d}{\varepsilon_0 \left(t + d\varepsilon_r\right)} \tag{S8}$$

Here, d is the distance of two triboelectric layers. t is the thickness of the dielectric film, σ is the charge density of the triboelectric layers.

Therefore, if we want to avoid the air breakdown, the voltage between two triboelectric layers must be smaller than the limited breakdown voltage calculated by Paschen's law, as shown in Equation (1).

$$V_b \geq V_{1S\text{[illegible]}} \tag{S9}$$

$$\frac{Bpd}{\ln(Apd) - \ln(\ln(1+\frac{1}{\gamma_{se}}))} \geq \frac{t\sigma d}{\varepsilon_0 (t + d\varepsilon_r)} \tag{S10}$$

$$\sigma \leq \frac{Bp\varepsilon_0 (t + d\varepsilon_r)}{t(\ln(Apd) + \ln(\ln(1+\frac{1}{\gamma_{se}})))} \tag{S11}$$

for all equation $d \in [0, d_{max}]$. Therefore, if we want to get the charge density to ensure that there was no breakdown during the whole moving process, the charge density should be smaller than the minimum of the right part of the equal sign in Equation (S12). And then we can easily get that the maximum charge density is equal to this minimum value, as shown in Equation (S13).

$$\sigma \leq \min\left[\frac{Bp\varepsilon_0 (t + d\varepsilon_r)}{t\left(\ln(Apd) + \ln(\ln(1+\frac{1}{\gamma_{se}}))\right)}\right] \tag{S12}$$

$$\sigma_{max} = \min\left[\frac{Bp\varepsilon_0 \left(t + d\varepsilon_r\right)}{t\left(\ln\left(Apd\right) + \ln\left(\ln\left(1+\frac{1}{\gamma_{se}}\right)\right)\right)}\right] \tag{S13}$$

Note S4 The method to achieve the higher transferred charge.

One tip to get the higher charge density is decreasing the contact area, because the larger area usually means the higher percentage of incomplete contact. But in some degree, small area will hardly accumulate high enough charge in the short time. In order to solve this, we use the self-enhancing circuit, proposed by Liu. [4] The electric circuit diagram is shown in Figure S4a and S4b. When the potential of the point 2, #2, is over than that of point 1, #1, the positive charge will be accumulated in the bottom electrode. And inversely, the charge accumulated in the bottom before will charge the capacitor and the top electrodes, which can help TENG cause more triboelectric charges. These two processes continue alternately until the amount of charge generated reaches the device's breakdown limit. Compared with the initial data of one CS TENG, as shown in Figure S4c and S4d, we can clearly find that the data of the experiment with the self-enhancing circuit has been improved highly and can reach its limited value in a short time.

Note S5 The method to calculate the energy limited by the breakdown effect.

In order to get the energy of TENG limited by the breakdown effect, we need to plot the *V-Q* curve for the CMEO cycle. Firstly, we fixed the displacement, d, of TENGs at a certain value, and then change the transferred charge of TENGs at this state from 0 to the maximum short-circuit transferred charge, to simulate all the states of the device from the open-circuit to short-circuit. By comparing the voltage distribution or the electric field distribution with the limited value, we can Figure out the breakdown voltage and charge of the device at this certain displacement. In the experiment, this is the process to calculate the breakdown voltage and charge as shown in Figure 2. Fixed the device at a fixed distance, and then increase its voltage and charge by high-voltage source until the breakdown happens. And then plot these values in the *V-Q* curve. By using the same method, we can calculate all the corresponding breakdown voltage and charge for every displacement. Finally plot the complete *V-Q* curve for this device. As shown in Figure S7a and S7b, each dotted line represents the transferred charge and the voltage of the TENGs at a fixed displacement. Due to capacitance of CS TENG is more variable than SFT TENG with a dielectric top layer, and thereby its slopes of each grey dotted lines are more variable than that of SFT TENG. And red points are the threshold breakdown voltage and charge for TENGs. When the voltage is over it or the transferred charge is smaller than that, the breakdown happens and the energy in this part cannot be used. Hence, the energy limited by the breakdown in this *V-Q* curve is the white area in this diagram. We can integrate the boundary curve to get the area of the white part we need. It is worth noting that, as shown in Figure S7c for CS TENG, we use the sum of S_1, S_2 and S_3 as the maximum energy limited the breakdown effect, without S_4. Because although the S_4 is white, in practical applications, before reach this voltage the device has already breakdown at the smaller voltage[1a]. In the same way, we can divide the white area in Figure S7d for SFT TENG into four parts and calculate the integral area of each part [1b].

[1] a) X. Xia, J. Fu, Y. Zi, *Nat. Commun.* **2019**, 10, 1; b) J. Fu, X. Xia, G. Xu, X. Li, Y. Zi, *ACS Nano* **2019**, 13, 13257.

[2] a) S. W. Niu, Sihong , Lin, Long , Liu, Ying , Zhou, Yu Sheng , Hu, Youfan , Wang, Zhong Lin, *Energy Environ. Sci.* **2013**, 6, 3576; b) S. Niu, Z. L. Wang, *Nano Energy* **2015**, 14, 161.

[3] Y. Zi, Wu, Changsheng, Ding, Wenbo, Wang, Zhong Lin, *Adv. Funct. Mater.* **2017**, 27,

1700049.

[4] W. Liu, Z. Wang, G. Wang, G. Liu, J. Chen, X. Pu, Y. Xi, X. Wang, H. Guo, C. Hu, Z. L. Wang, *Nat. Commun.* **2019**, 10, 1426.
